# Trypanocidal Activity of Oxoaporphine and Pyrimidine-β-Carboline Alkaloids from the Branches of *Annona foetida* Mart. (Annonaceae)

**DOI:** 10.3390/molecules16119714

**Published:** 2011-11-23

**Authors:** Emmanoel Vilaça Costa, Maria Lúcia Belém Pinheiro, Afonso Duarte Leão de Souza, Andersson Barison, Francinete Ramos Campos, Rodrigo Hinojosa Valdez, Tânia Ueda-Nakamura, Benedito Prado Dias Filho, Celso Vataru Nakamura

**Affiliations:** 1 LABORGANICS, Department of Chemistry, Federal University of Sergipe, 49100-000, São Cristóvão, SE, Brazil; Email: emmanoelvilaca@yahoo.com.br (E.V.C.); 2 Department of Chemistry, Federal University of Amazonas, 69077-000, Manaus, AM, Brazil; 3 Department of Chemistry, Federal University of Paraná, 81531-990, Curitiba, PR, Brazil; Email: andernmr@ufpr.br (A.B.); 4 Department of Pharmacy, Federal University of Paraná, 80210-170, Curitiba, PR, Brazil; Email: francampos@ufpr.br (F.R.C.); 5 Department of Basic Health Sciences, State University of Maringá, 87020-900, Maringá, PR, Brazil; Email: valdez.rodrigo@gmail.com (R.H.V.); tunakamura@uem.br (T.U.-N.); bpdfilho@uem.br (B.P.D.F.); cvnakamura@uem.br (C.V.N.)

**Keywords:** annonaceae, *Annona foetida*, alkaloids, oxoaporphine alkaloids, pyrimidine-β-carboline alkaloids, trypanocidal activity

## Abstract

Phytochemical investigation of the branches of *Annona foetida* Mart. led to isolation from the CH_2_Cl_2_ extract of four alkaloids: Atherospermidine (**1**), described for the first time in this species, liriodenine (**2**), *O*-methylmoschatoline (**3**), and annomontine (**4**). Their chemical structures were established on the basis of spectroscopic data from IR, MS, NMR (1D and 2D), and comparison with the literature. Compounds **2**–**4** showed potent trypanocidal effect when evaluated against epimastigote and trypomastigote forms of *Trypanosoma cruzi*.

## 1. Introduction

*Annona* L. belongs to the family Annonaceae and comprises approximately 175 species of trees and shrubs found predominantly in lowland tropical regions [1]. Economically, this genus is the most important of the Annonaceae family due to its edible fruits and medicinal properties [2]. Previous chemical and pharmacological investigations on some species of this genus have indicated the presence of important bioactive compounds, exhibiting various pharmacological activities including cytotoxicity against various tumor cell lines [3,4,5], antimicrobial [6,7], antioxidant [6], antiplatelet [8,9], and antiparasitic properties, in particular against *Leishmania* sp. [7,10,11,12] and *Trypanosoma cruzi* [10,12,13]. These activities generally are attributed to the presence of alkaloids, acetogenins, and terpenes. In Brazil this genus contain close to 60 species, with the largest part occurring in forests and few representatives in open areas.

*Annona foetida* Mart. is a 3–15 m tall tree found in the Brazilian and Peruvian Amazon region. It is popularly known as “envira-ata”, “envireira”, “araticum caatinga” and “graviola da mata” [2,14]. In folk medicine the leaves and bark decoction are used for treatment of rheumatism, intermittent fevers and ulcers [2]. Previous studies on this species describe the isolation of oxoaporphine and pyrimidine-β-carboline alkaloids [11], and essential oils with antileishmanial and antimicrobial activities [7]. As a result of our continuing study on *A. foetida* in a search for antiparasitic natural products, mainly against *T. cruzi*, we report herein the phytochemical and biological study of the CH_2_Cl_2_ extract from the branches of this species.

## 2. Results and Discussion

**Figure 1:**
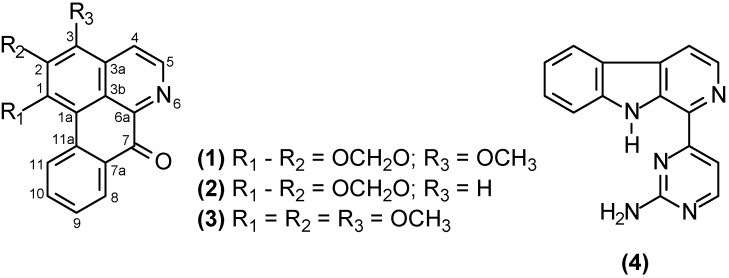
Alkaloids isolated from the branches of *A. foetida*.

Phytochemical investigation of CH_2_Cl_2_ extract from the branches of *Annona foetida* led to the isolation of four alkaloids (Figure 1), namely three oxoaporphine atherospermidine (**1**), liriodenine (**2**) and *O*-methylmoschatoline (**3**) and one pyrimidine-β-carboline one, annomontine (**4**).

The structural elucidation of these alkaloids was carried out based on their spectroscopic data, mainly 1D and 2D NMR, and also comparison with the literature data [11,13,15,16,17]. Atherospermidine (**1**) is described for the first time in this species, while compounds **2**–**4** have been described in the bark. Atherospermidine (**1**), liriodenine (**2**), and *O*-methylmoschatoline (**3**) are three oxoaporphine alkaloids widely found in almost all the genera of the family Annonaceae. However, the pyrimidine-β-carboline alkaloid annomontine (**4**) has been described only in the genus *Annona* [11,18,19]. Natural products or derivatives play an important role in the development of all types of drugs and some natural compounds or extracts have shown trypanocidal activity [20]. The compounds liriodenine (**2**), *O*-methylmoschatoline (**3**), and annomontine (**4**) were investigated for their biological activity against epimastigote and trypomastigote forms of *T. cruzi* (Table 1). The comparison of the 50% inhibitory concentration values (IC_50_ in µg/mL) revealed that all three evaluated compounds showed some activity against epimastigote forms, with *O*-methylmoschatoline (**3**) being the most active isolated compound, with an IC_50_ value of 92.0 ± 18.4 µg/mL. The presence of **3** in the culture of epimastigotes caused progressive parasite injury, compared to untreated cells, and a dose-dependent effect was also observed. After 96 h of incubation with the concentration of 500 µg/mL, parasite growth was completely arrested.

**Table 1:** Trypanocidal activity of the oxoaporphine and pyrimidine-β-carboline alkaloids (**2**–**4**).

Compounds	IC_50_ (µg/mL)	EC_50_(µg/mL)
	Epimastigote forms	Trypomastigote forms
Liriodenine (**2**)	177.0 ± 10.6	4.0 ± 0.2
*O*-methylmoschatoline (**3**)	92.0 ± 18.4	3.8 ± 1.8
Annomontine (**4**)	198.0 ± 4.2	4.2 ± 1.9
Benznidazole ^a^	2.0 ± 0.9	
Crystal violet ^b^		12.8± 0.9

^a,b^ Positive controls against epimastigote and trypomastigote forms of *T. cruzi*.

Besides, the evaluated compounds **2**–**4** showed a high level of lytic activity over trypomastigote forms of *T. cruzi*. On a dose-dependent trypanocidal effect experiment, all of them were more active than the positive control crystal violet (Table 1). While this reference drug has an EC_50_ of 12.8 ± 2.6 µg/mL against trypomastigote forms of *T. cruzi*, the best activity, showed by *O*-methylmoschatoline (3) was of EC_50_ of 3.8 ± 1.8 µg/mL in the presence of mouse blood. Biological activity of compounds **2**–**4** has also been described for other protozoans such as *Leishmania braziliensis* and *L. guynensis* [11]. Moreover, regarding the importance of its biological evaluation other aporphine and β-carboline alkaloids showed trypanocidal activity over *T. cruzi* [21,22].

## 3. Experimental

### 3.1. General

UV spectra were obtained in CH_3_OH on a UV-Vis Agilent HP 8453 spectrophotometer. IR spectra were acquired on a Bomem MB-100 spectrophotometer. 1D and 2D NMR experiments were acquired in CDCl_3_, CDCl_3_ + CD_3_OD or CD_3_OD at 293 K on a Bruker AVANCE 400 NMR spectrometer operating at 9.4 Tesla, operating at 400 and 100 MHz for ^1^H and ^13^C at, respectively. The spectrometer was equipped with a 5-mm multinuclear direct detection probe with *z*-gradient. One-bond and long-range ^1^H-^13^C correlation (HSQC and HMBC) experiments were optimized for an average coupling constant ^1^*J*_(C,H)_ and ^LR^*J*_(C,H)_ of 140 and 8 Hz, respectively. All ^1^H- and ^13^C-NMR chemical shifts (*δ*) are given in ppm related to the TMS signal at 0.00 ppm as internal reference, and the coupling constants (*J*) in Hz. Low-resolution ESI-MS data were taken in the positive ion mode, on a Micromass Quattro LC mass spectrometer. Silica gel 60 (70–230 mesh) was used for column chromatography, while silica gel 60 F_254_ were used for analytical (0.25 mm), and preparative (1.00 mm) TLC. Compounds were visualized by exposure under UV_254/366_ light, spraying *p*-anisaldehyde reagent followed by heating on a hot plate, as well as spraying with Dragendorff’s reagent.

### 3.2. Plant Material

The branches of *Annona foetida* were collected from Adolpho Ducke Reserve [coordinates: 02° 54' 26'' to 03° 00' 22'' S, 59° 52' 40'' to 59° 58' 40'' W], close to Manaus city, Amazonas, Brazil, in August 2002. Its identification was done by Dr. Antonio Carlos Webber, a plant taxonomist of the Biology Department of the Federal University of Amazonas (UFAM), and a voucher specimen (#7275) has been deposited in the Herbarium of this University in Manaus, Amazonas, Brazil.

### 3.3. Extraction and Isolation Procedures

Dried and powdered branches of *A. foetida* (1,900 g) were successively extracted with *n*-hexane, CH_2_Cl_2_ and MeOH (4.5 L, four times for each solvent), to yield hexane (6.07 g), CH_2_Cl_2_ (9.43 g) and MeOH (50.0 g) extracts after solvent removal. TLC investigations indicated a high concentration of alkaloids in the CH_2_Cl_2_ extract. A part of this extract (9.20 g) was initially subjected to an acid-base extraction [11] to give CH_2_Cl_2_ alkaloid (0.54 g) and CH_2_Cl_2_ neutral fractions (8.08 g). The alkaloid fraction (0.50 g) was subjected to a 10% NaHCO_3_ treated silica gel column chromatography [11] eluted with the gradient systems: hexane-CH_2_Cl_2_ from 100:0 to 10:90, followed by CH_2_Cl_2_-EtOAc from 100:0 to 10:90, and EtOAc-MeOH from 100:0 to 50:50 yielding 78 subfractions. The eluted subfractions were evaluated and pooled according to TLC analysis, to afford 12 groups. Group 5 (140.0 mg) was purified by preparative TLC eluted with hexane-acetone (60:40, three times) affording **1** (1.5 mg), **2** (17.0 mg) and **3** (6.5 mg), respectively. Group 7 (39.0 mg) was also purified by preparative TLC eluted with CH_2_Cl_2_-MeOH (95:05, twice) resulting in **4** (7.2 mg).

*Atherospermidine* (**1**): Orange crystals (CH_2_Cl_2_:MeOH 3:1); mp 286–287 °C (lit. 284–286 °C) [15]; UV (CHCl_3_) λ_max_/nm 250, 286, 315, 384; IV (Film, CHCl_3_) ν_max_/cm^−1^ 1656, 1070, 847, 755; ^1^H-NMR (CDCl_3_) δ 8.88 (1H, d, *J* = 5.3 Hz, H-5), 8.50 (1H, dd, *J* = 8.2 and 1.5 Hz, H-11), 8.52 (1H, dd, *J* = 8.1 and 1.5 Hz, H-8), 8.11 (1H, d, *J* = 5.3 Hz, H-4), 7.67 (1H, ddd, *J* = 8.2, 7.2 and 1.5 Hz, H-10), 7.47 (1H, ddd, *J* = 8.0, 7.2 and 1.5 Hz, H-9), 6.30 (2H, s, 1-OCH_2_O-2), 4.28 (3H, s, H_3_CO-3); ^13^C-NMR (CDCl_3_) δ 182.6 (C-7), 149.5 (C-1), 144.9 (C-6a), 144.3 (C-5), 136.3 (C-2), 136.1 (C-3), 134.1 (C-10), 133.1 (C-11a), 130.61 (C-7a), 130.64 (C-3a), 128.5 (C-8), 127.4 (C-9), 126.7 (C-11), 122.8 (C-3b), 119.5 (C-4), 102.6 (C-1a), 102.3 (1-OCH_2_O-2), 60.2 (H_3_CO-3); ESI-MS *m/z *306.2 [M+H]^+^.

*Liriodenine* (**2**): Yellow crystals (CH_2_Cl_2_:MeOH 3:1); mp 281–282 °C (lit. 280–281 °C) [11]; UV (CHCl_3_) λ_max_/nm 208, 220 (sh), 246, 268, 302 (sh), 414; IV (Film, CHCl_3_) ν_max_/cm^−1^ 2922, 2852, 1654, 1596, 1577, 1485, 1470, 1422, 1443, 1384, 1311, 1262, 1228, 1207, 1117, 1051, 1017, 964, 911, 872, 779, 752, 725, 690, 609, 570; ^1^H-NMR (CDCl_3_) δ 8.87 (1H, d, *J* = 5.2 Hz, H-5), 8.61 (1H, ddd, *J* = 8.1, 1.0 and 0.5 Hz, H-11), 8.57 (1H, ddd, *J* = 7.9, 1.4 and 0.5 Hz, H-8), 7.75 (1H, d, *J* = 5.2 Hz, H-4), 7.73 (1H, ddd, *J* = 8.1, 7.4 and 1.4 Hz, H-10), 7.56 (1H, ddd, *J* = 7.9, 7.4 and 1.0 Hz, H-9), 7.16 (1H, s, H-3), 6.37 (2H, s, 1-OCH_2_O-2). The ^13^C-NMR data are in agreement with the literature [13,16]; ESI-MS *m/z* 276.0 [M+H]^+^.

*O*-*Methylmoschatoline* (**3**): Orange crystals (CH_2_Cl_2_:MeOH 2:1); mp 182–183 °C (lit. 181–182 °C) [11]; UV (CHCl_3_) λ_max_/nm 242, 272, 311 (sh), 428; IV (Film, CHCl_3_) ν_max_/cm^−1^ 2937, 2853, 1663, 1595, 1579, 1472, 1392, 1311, 1257, 1205, 1158, 1117, 1094, 1043, 1007, 973, 940, 769, 691; ^1^H-NMR (CDCl_3_) δ 9.11 (1H, ddd, *J* = 8.4; 1.1, and 0.6 Hz, H-11), 9.00 (1H, d, *J* = 5.3 Hz, H-5), 8.57 (1H, dd, *J* = 7.9 and 1.4 Hz, H-8), 8.24 (1H, d, *J* = 5.3 Hz, H-4), 7.75 (1H, ddd, *J* = 8.4, 7.2 and 1.5 Hz, H-10), 7.54 (1H, ddd, *J* = 7.9, 7.2 and 1.1 Hz, H-9), 4.20 (3H, s, H_3_CO-3) 4.11 (3H, s, H_3_CO-2), 4.08 (3H, s, H_3_CO-1); ^13^C-NMR (CDCl_3_) δ 182.6 (C-7), 156.4 (C-1), 148.4 (C-3), 147.3 (C-2), 145.4 (C-6a), 144.5 (C-5), 134.5 (C-11a), 134.3 (C-10), 131.4 (C-7a), 131.1 (C-3a), 128.9 (C-8), 128.1 (C-9), 127.6 (C-11), 122.8 (C-3b), 119.1 (C-4), 115.6 (C-1a), 61.8 (3- H_3_CO-3), 61.4 (H_3_CO-2), 61.0 (1- H_3_CO-1); ESI-MS *m/z* 322.6 [M+H]^+^.

*Annomontine* (**4**): Yellow crystals (CH_2_Cl_2_:MeOH 3:1); mp 248–249 °C (lit. 249–250 °C) [11]; identified by comparison with literature data (co-TLC, mp, UV, IR, MS, ^1^H-NMR and ^13^C-NMR) [11,17].

### 3.4. In Vitro Trypanocidal Assay

*Parasites:* Epimastigote forms of *Trypanosoma cruzi* strain Y were grown in Liver Infusion Tryptose (LIT) supplemented with 10% fetal-calf serum (FCS, Gibco) at 28 °C for 96 h.

*Cell culture:* LLCMK_2_ (monkey kidney cells) were maintained in DMEM supplemented with 2 mM L-glutamine, 10% FCS, and 50 mg·L^−1^ gentamycin, and buffered with sodium bicarbonate.

*Antiproliferative activity on epimastigote forms:* Epimastigote forms of *T. cruzi* in the logarithmic phase were used for this assay. The compounds liriodenine (**2**), *O*-methylmoschatoline (**3**) and annomontine (**4**) were dissolved in DMSO and LIT medium to obtain concentrations of 1, 5, 10, 50, 100, 500 and 1,000 µg/mL, in different wells. The final concentration of DMSO did not exceed 1%.

A cell density of 1 × 10^6^ epimastigotes/mL was cultured in a 24-well microplate to obtain a final volume of 1 mL. The cells were incubated at 28 °C and their growth was determined by counting the parasites with a hemocytometer chamber after 96 h. Benznidazole was used as the reference drug. The IC_50_ value (50% inhibitory concentration) was determined using linear regression analysis from this inhibitory percentage. These tests were performed in triplicate on separate occasions.

*Activity against trypomastigote forms:* The tissue-culture-derived trypomastigotes were resuspended in Dulbecco’s modified Eagle medium supplemented with FCS containing 10% mouse blood in a concentration of 10^7^ parasites/mL. In a 96-well microplate, 100 µL of this suspension was added to the same volume of the compounds diluted in DMSO and DMEM at twice the desired final concentration (1, 5, 10, 50, 100, 500 and 1000 µg/mL), and incubated for 24 h at 37 °C. We used the Pizzi-Brener method to appreciate the mobility, thus the viability of this form of parasite. For this an aliquot of 5 µL of each sample were placed on slides plus coverslips and immediately counted in an optical microscopy [23], subsequently the EC_50_ (concentration which lysed 50% of the parasites) was calculated. Crystal violet was used as the reference drug.

## 4. Conclusions

This work resulted in the isolation and identification of three oxoaporphine [atherospermidine (**1**), liriodenine (**2**) and *O*-methylmoschatoline (**3**)] and one pyrimidine-β-carboline alkaloid [annomontine (**4**)] from the branches of *Annona foetida*, compound **1** being described for the first time in this species. These finds contribute to the chemotaxonomy of the family Annonaceae, especially for the genus *Annona*. Trypanocidal activity assays revealed that all alkaloids tested (compounds **2**–**4**) shown strong trypanocidal effects against the clinical relevant trypomastigote forms of *T. cruzi*, even better than positive control. The oxoaporphine alkaloid *O*-methylmoschatoline (**3**) was the most active for both epimastigote and trypomastigote forms. This results support further evaluations over analogs, as well as encourages *in vitro* and *in vivo* investigations of these alkaloid kinds.
